# Are Upper-Body Axial Symptoms a Feature of Early Parkinson’s Disease?

**DOI:** 10.1371/journal.pone.0162904

**Published:** 2016-09-21

**Authors:** Caroline Moreau, David Devos, Guillaume Baille, Arnaud Delval, Céline Tard, Thierry Perez, Nicolas Danel-Buhl, David Seguy, Julien Labreuche, Alain Duhamel, Marie Delliaux, Kathy Dujardin, Luc Defebvre

**Affiliations:** 1 Department of Neurology and Movement Disorders, CHU, Lille, France; 2 Department of Medical Pharmacology, Disorders, CHU, Lille, France; 3 Department of Clinical Neurophysiology, Disorders, CHU, Lille, France; 4 Department of Pulmonology, CHU, Lille, France; 5 Department of Nutrition, Lille CHU, Lille, France; 6 Department of Biostatistics, CHU, Lille, France; 7 INSERM UMR_S 1171, Lille, France; 8 University of Lille, Lille, France; University of Tuebingen, GERMANY

## Abstract

**Background:**

Axial disorders are considered to appear late in the course of Parkinson’s disease (PD). The associated impact on quality of life (QoL) and survival and the lack of an effective treatment mean that understanding and treating axial disorders is a key challenge. However, upper-body axial disorders (namely dysarthria, swallowing and breathing disorders) have never been prospectively assessed in early-stage PD patients.

**Objectives:**

To characterize upper-body axial symptoms and QoL in consecutive patients with early-stage PD.

**Methods:**

We prospectively enrolled 66 consecutive patients with early-stage PD (less than 3 years of disease progression) and assessed dysarthria, dysphagia and respiratory function (relative to 36 controls) using both objective and patient-reported outcomes.

**Results:**

The mean disease duration was 1.26 years and the mean UPDRS motor score was 19.4 out of 108. 74% of the patients presented slight dysarthria (primarily dysprosodia). Men appeared to be more severely affected (i.e. dysphonia). This dysfunction was strongly correlated with low swallowing speed (despite the absence of complaints about dysphagia), respiratory insufficiency and poor QoL. Videofluorography showed that oral-phase swallowing disorders affected 60% of the 31 tested patients and that pharyngeal-phase disorders affected 21%. 24% of the patients reported occasional dyspnea, which was correlated with anxiety in women but not in men. Marked diaphragmatic dysfunction was suspected in 42% of the patients (predominantly in men).

**Conclusion:**

Upper body axial symptoms were frequent in men with early-stage PD, whereas women presented worst non-motor impairments. New assessment methods are required because currently available tools do not reliably detect these upper-body axial disorders.

## Introduction

Parkinson’s disease (PD) is a neurodegenerative disorder that affects 1–2% of the world’s population [1]. A turning point in the course of the disease is the occurrence of cognitive and axial symptoms. Of these, gait disorders and falls have a major impact on disease progression, the patient’s quality of life (QoL), and the caregiver burden [2,3].

Here, we prospectively assessed both lower-body axial signs (gait disorders, freezing and postural instability) and upper-body axial signs (namely dysarthria, deglutition and respiratory function) in a cohort of 66 consecutive PD patients. In particular, we sought to define the frequency and nature of upper-body axial signs.

Dysarthria is a common, well-described feature of advanced PD, with dysprosodia and hypophonia as hallmark features [4,5]. A retrospective study found that dysarthria appears 84 months after disease onset, on average [2]. However, impaired phonation can occur very early in the course of the disease, with hoarseness, hypophonia and/or tremulousness associated with incomplete closure of the vocal folds [6]. Some patients even report voice abnormalities before the onset of motor symptoms [7]. Moreover, speech rate and rhythm appear to be reliable markers of disease progression, since they are well correlated with the occurrence of axial and gait disorders [8,9].

It has been estimated that dysphagia and its complications (i.e. lung infections) occur after a mean period of 130 months after disease onset. These disorders are associated with a significant reduction in the survival time; death typically occurs within 15 to 24 months [2].

Literature studies of respiratory insufficiency and muscle weakness in advanced PD have suffered from a number of limitations: small study populations, predominantly late-stage PD, a lack of long-term follow-up and a lack of functional evaluations in the “off-drug” condition. Although there are many reports of abnormal spirometry data in advanced PD patients, little is known about respiratory function in early PD.

Dysarthria, dysphagia and breathing are interlinked, since (i) hypophonia results from an abnormally low expiratory pressure and (ii) dysphagia (aspiration) is caused by impaired coordination of the upper airways during swallowing—namely a prolonged swallow phase during inspiration in PD patients [10].

There is a need for early clinical biomarkers of axial symptoms. Here, we prospectively enrolled 66 consecutively early-stage PD patients (including 39 *de novo*, drug-naïve patients) in a clinical, functional and QoL assessment of upper axial signs (i.e. dysarthria, swallowing and breathing). Our hypothesis was that upper axial signs are relatively frequent and underdiagnosed in early-stage PD. If these signs occur very early in the disease process, they may contribute to the motor and cognitive prognosis.

## Subjects and Methods

### Subjects

Seventy PD patients were consecutively enrolled between 2011 and 2013. The inclusion criteria were as follows: patients meeting the United Kingdom Parkinson’s Disease Society Brain Bank diagnostic criteria [11], normal MRI, a DaTscan confirming dopaminergic depletion, and the absence of respiratory tract, ear, nose or throat disease. The main exclusion criteria were dementia (according to DSM V) and inability to give informed consent.

All patients had undergone 3T MRI and DaTscan imaging at the time of diagnosis, in order to confirm dopaminergic depletion and rule out vascular lesions. At that time, two patients were ruled out because of repeated normal DaTscans. Moreover, we checked the patients’ diagnostic criteria in late 2014 and 2015, in order to exclude any participants with atypical PD. Indeed, we excluded two further patients at that time because they were respectively diagnosed with multiple system atrophy and progressive supranuclear palsy. The present study was approved by the local investigational review board (*CPP Nord-Ouest IV*, Lille, France; approval number: 2010-A01391-38) and registered on the ClinicalTrials.gov website (NCT 02627664). All the subjects gave their written, informed consent.

Thirty-six controls (matched for age and gender with the PD patients) also underwent pulmonary function testing (PFT). None of the controls presented with neurologic disease. To increase comfort, safety and reproducibility, PD patients were asked to attend the outpatient clinic twice. During the first visit, patients were clinically evaluated in the “off-drug” condition (i.e. at least 12 hours after the withdrawal of levodopa treatment). They then completed the self-questionnaires and the neuropsychological evaluation in the afternoon (in the “on-drug” condition). During the second visit (in the morning), the patients underwent PFT and a videofluorographic assessment of swallowing in the “off-drug” condition.

### Clinical assessments

An experienced neurologist assessed motor aspects of PD. Neuropsychological evaluations included the Mini Mental State Examination, the Montreal Cognitive Assessment, the Mattis Dementia Rating Scale (in order to exclude demented patients), the Lille Apathy Rating Scale, the Montgomery-Asberg Depression Rating Scale (MADRS) and the Hamilton Anxiety Rating Scale (HAM-A).

### Speech assessments

***A perceptive analysis*** was performed by an experienced speech therapist, using part of the validated *Batterie Clinique d’Évaluation de la Dysarthrie* (BECD, a French adaptation of the Frenchay Dysarthria Assessment) [12]. We applied a composite scale for qualitative speech analysis (range: 0 to 140) comprising six subscores: quality of voice, phonetic realization, dysprosody, intelligibility, speech intonation, and respiration.

***An acoustic analysis*** of speech intensity, maximum phonation time, fundamental frequency (F0) and the latter’s coefficient of variation (CVF0) was performed with a computer-assisted voice analysis tool (EVA 2, *Evaluation Vocale Assistée 2*, SQLAB, Aix-en-Provence, France). Maximum phonation time and speech intensity were calculated during a sustained /a/. Prosody (CVF0), jitter, shimmer and harmonic-to-noise ratios were measured while the patient read a standard text.

The speech analysis data were analyzed by gender and compared with our laboratory’s reference dataset (49 female controls and 50 male controls with no evidence of neurodegenerative disease and no family links to the PD patients; mean ± SD age: 61.2 ± 6.9) [5].

### Swallowing assessments

We assessed deglutition with (i) the Deglutition Handicap Index (DHI) self-questionnaire, (ii) a timed swallowing test with a glass of water (previously validated in patients with neurologic disease) [13], and (iii) an objective, blind, videofluorographic evaluation in the “off-drug” condition by two experienced operators.

In the timed swallowing test, the subject was asked to drink a 150 mL glass of cold tap water while seated. The required time (in seconds), swallowed volume (in mL), swallowing speed (in mL/s), any coughing during or after the test and the voice quality after the test were noted. We compared the patient data with the above-mentioned control data [13].

Videofluorography was always performed by the same two operators. The assessment was performed in the off-drug condition. Patients were asked to swallow a 10 mL bolus of low-density barium (40% w/v). The assessment was performed twice. Side and frontal views were examined. A single-blind analysis by the two experienced evaluators focused on qualitative impairments of the oral phase (side view) and the pharyngeal phase (side and frontal views) and the presence or absence of aspiration.

### Pulmonary function testing

PFT was performed at 8.30 am (in the “off-drug” condition) using impulse oscillometry (Masterscreen IOS, Becton Dickinson, UK). We recorded the forced vital capacity, vital capacity (VC, measured by slow exhalation), the peak expiratory flow rate and the forced expiratory volume in one second (FEV1). Muscle strength was assessed (using the HypAir system from Medisoft, Sorinnes, Belgium) in terms of the maximal inspiratory pressure (MIP), the sniff nasal inspiratory pressure and the maximal expiratory pressure (MEP). PFT was performed in line with the European Respiratory Society guidelines [14]. Individual analyses were performed according to Uldry et al.’s method [15]. Magnetic stimulation of the phrenic nerve was performed if inspiratory weakness was detected (in order to highlight any central nervous system involvement).

The patients’ results were compared with those from 36 healthy controls (mean ± SD age: 61.1 ± 4.1). None of the controls had signs of neurodegenerative disease or pulmonary dysfunction, and none were related to the PD patients.

### Self-questionnaires

Validated self-questionnaires (including the Movement Disorder’s Society UPDRS part I and II for cognitive, psychological and non-motor aspects of the disease) were administered to each patient [16].The validated French version of the **Voice Handicap Index** (VHI) [17] was used to assess the physical, functional and emotional consequences of speech impairment. The maximum score is 80 and a cut-off of 20 is used to distinguish between dysphonic and non-dysphonic subjects [17]. The validated French version of the **Deglutition Handicap Index** (DHI) [18] is divided into three 10-item domains (physical, functional and emotional). The maximum score is 120, and the DHI’s physical domain is a highly sensitive guide to the severity of deglutition disorders [18]. The **Medical Research Council** (MRC) breathlessness scale was used to determined self-reported dyspnea [19]. The score was subsequently dichotomized (score of 1: no dyspnea; score of 2 to 5: dyspnea). In order to assess possible bias associated with somatic manifestations of anxiety, we analyzed the correlations between the MRC dyspnea score on one hand and the MDS UPDRS I anxiety subscore (item 4) and the HAM-A cardiovascular/respiratory subscore (items 9 and 10) on the other.

### Statistical analysis

There are no literature data on the frequency of axial disorders in *de novo*, early PD patients. However, our previous research results [4,5] suggest that 25% of the patients at this stage of the disease have severe dysarthria. Hence, we sought to recruit 70 patients and thus obtain between 10 and 15 patients with severe dysarthria (with intelligibility defined as a score >2 in item 18 of the UPDRS part III).

Baseline characteristics were described as the mean ± standard deviation (SD). After checking the Gaussian distribution of the data with the Kolmogorov-Smirnov Test, intergroup comparisons were performed with a T-test. For non-normally distributed data or comparisons between small samples, we applied a Mann-Whitney test for continuous variables and a chi-squared test for categorical variables. Fisher’s exact test was used to compare frequencies. In view of our study’s exploratory nature and small sample size, we did not adjust for multiple comparisons. Pearson’s correlation coefficient was calculated. The threshold for statistical significance was set to p<0.05 in all tests. All analyses were performed with SPSS for Windows software (version 22.0, IBM Inc., Armonk, NY, USA).

## Results

The results appeared in Table 1. 39 *de novo* patients had experienced their first symptoms about 1 year previously. The 27 treated patients had experienced their first symptoms less than 3 years previously. The mean equivalent levodopa dose was 371.05 ± 132.06 mg/day (dopamine agonists: 56%; levodopa: 44%). 66% of the treated patients were receiving rasagiline (1 mg per day).

**Table 1:** Baseline demographic data for the PD patients enrolled in the study.

	All PD patients	*De novo* patients	Treated patients	Female patients	Male patients	PIGD patients	TD patients	Akinetic- rigid phenotype
n	66	39	27	24	42	19	19	28
Age (years)	62.5 (7.9)	62.8 (6.7)	62 (10)	62.1 (8)	62.6 (7)	63.06 (7.8)	61.8 (8.1)	62.7 (7.8)
Disease duration (years)	1.26 (1.0)	0.64 (0.4)	2.59 (0.5)	1.35 (1.1)	2.16 (1.5)	1.09 (1.0)	1.55 (1.0)	1.6 (1.4)
Males/females	42/24	22/17	21/7	24	42	14/5	8/11	17/11
MMSE	28.9 (1.8)	28.6 (1.9)	29.4 (1.4)	29.1 (1,9)	28.9(1.5)	29 (1.4)	28.6 (2.3)	28.4 (1.8)
MoCA	26.6 (2.7)	26.3 (3.2)	27.2 (1.8)	26.1 (3.2)	27.2 (2.0)	26.4 (3.1)	26.8 (2.2)	26.9 (3.1)
LARS	-26.3 (5.1)	-25.8 (5.2)	-27.4 (4.9)	-27.3 (4.9)	-26.5 (5.4)	-25.5 (5.2)	-27.7 (4.7)	-26.9 (3.1)
HAM-A	6.9 (6.0)	7.2 (6.1)	6.3 (6.0)	**8.6 (5.7)** *	5.77 (5.6)*	7 (6.0)	6.85 (5.8)	5.8(5.4)
MADRS	5.6 (5.3)	6.0 (5.5)	4.5 (4.9)	6.4 (5.2)	5.4 (5.5)	5.7 (5.5)	5.4 (5.0)	5 (4.9)
MDS UPDRS part I	5.4 (4.0)	5.06 (4.1)	6.2 (3.6)	5.7 (3.6)	6.2 (4.6)	5.3(4.1)	7.4(4.9)	5.16 (3.5)
MDS UPDRS part II	5.2 (3.9)	5 (3.7)	5.8 (4.2)	5.6 (4.5)	6.8 (4.3)	6.3 (4.4)	6 (4.6)	6.1 (4.1)
UPDRS part III	19.4 (8.1)	18.8 (8.7)	20.5 (6.9)	20.7 (9.2)	20 (7.4)	19.9 (8.0)	21.2 (8.2)	20 (7.8)
Tremor subscore	2,3 (2,4)	1.8 (1.8)	2.8 (2.7)	2.3 (2.3)	2.3 (2.4)	1.8 (1.2)	4.8 (2.3)	1.1 (0.3)
Rigidity subscore	5,3 (2,9)	8.4 (3.2)	5.5 (2.2)	5.0 (3.3)	5.4 (2.7)	4.8 (3.5)	5.1 (2.1)	6 (2.6)
Axial subscore	2.5 (1.3)	2.7 (1.2)	2.5 (1.3)	2.3 (2.9)	2.5 (2.3)	3.2 (1.1)	2 (0.8)	2.5 (1.3)
Dysarthria (item 18)	0.9 (0.6)	0.9 (0.6)	0.9 (0.5)	0.7 (0.6)*	1.1 (0.6)*	0.8 (0.7)	0.95 (0.5)	0.9 (0.6)
UPDRS part IV	1.1 (2.2)	1.1 (3.4)	1 (1.2)	1 (1.2)	1.1 (1.2)	0.6 (1.1)	0.9 (1.5)	1.3 (4)
Timed SWS test	13.9 (4.4)	13.9 (4.6)	13.9 (4)	14.5 (3.2)	13 (3.9)	13.5 (3.5)	14.3 (4.6)	13.4 (3)

Values are expressed as the mean ± SD; n: number; MMSE: Mini Mental State Examination; MoCA: Montreal cognitive assessment; MADRS: Montgomery-Asberg Depression Rating Scale; LARS: Lille Apathy Rating Scale; HAM-A: Hamilton Anxiety Rating Scale; UPDRS: Unified Parkinson’s Disease Rating Scale; MDS UPDRS: Movement Disorders Society Unified Parkinson’s Disease Rating Scale; SWS: stand-walk-sit test; PIGD: postural instability and gait disorders; TD: tremor-dominant.

* indicates p<0.05, with bold numbers for the concerned variables and groups.

In this group of 66 consecutive early PD patients, the mean age was 62.5 years and the mean disease duration was 1.26 years. At that stage, none of the subjects presented cognitive deterioration, marked apathy or depression.

Non-motor symptoms were more prominent in female patients, who had a significantly higher mean HAM-A score. For motor symptoms, the mean UPDRS score was 19.4 out of 108: dysarthria was more frequent and more severe in the men than in the women.

The ratio between the mean UPDRS tremor score (8 items) and the mean UPDRS postural instability and gait difficulty (PIGD) score (5 items) was used to differentiate between tremor-dominant (TD) patients and those with PIGD: <1 = PIGD; 1 to 1.5 = akinetic; >1.5 = TD [20]. Twenty-eight percent of the patients presented a PIGD phenotype and 28% presented a tremor dominant phenotype. However, 42% of the patients could not be classified regarding this ratio. None of the patients presented motor fluctuations or gait impairments.

### Speech assessments

An analysis of the total VHI score and the physical subscore revealed marked **gender differences**: men reported a significantly more severe voice handicap than women (mean total VHI score: 27 in men and 16 in women; p = 0.036; **Table 2**).

**Table 2:** Dysarthria and deglutition assessments in 66 PD patients.

	All PD patients	Female patients	Female controls	p	Male patients	Male controls	p
**Number of patients**	66	24	49		42	50	
**Speech evaluation**							
VHI (out of 120)	26.3 (17)	16 (20)*	-		27.1 (30)*	-	0.036
VHI physical subscore (out of 40)	9.1	7.9 (8.8)*	-		11.3 (7)*	-	0.044
MDS UPDRS item 2.1		0.88 (0.7)	-		0.68 (0.7)	-	
BECD total score (out of 140)	3.9 (2.7)	3 (2.7)*	-		4.3(3.3)*	-	0.058
MPT (s)		13.9 (6)	13.8 (6)		16.4 (5)	17.5 (9)	
Mean intensity (db)		73.1 (6)	69 (5)		73.5 (6)	73.5 (6)	
Mean F0 (Hz)		214 (20)	196 (30)		134 (18)	115 (19)	
CVF0 (%)		20.5 (9)	30 (15)	0.043	18 (8)	29 (17)	0.037
**Deglutition assessments**								
MDS UPDRS item 2.2		0.32 (0.4)	-		0.48(0.6)	-	
MDS UPDRS item 2.3		0.37 (0.5)	-		0.36(0.6)	-	
DHI (out of 120)	9.9 (8.1)	8.8 (11.4)	-		10.1 (9)	-	
DHI physical subscore (out of 40)	4.9 (4.3)	4.4 (4.2)	-		5.8 (5.2)	-	
Swallowing speed (150 mL)		15.5 (7.55) ^§^	21 (4 .55) ^§^	0.03	20 (9.98)^+^	29 (8.3)^+^	0.029
**Videofluorography**								
Oral-phase abnormalities n (%)	19 (60)	9 (30)	-		10 (63)	-	
Uncontrolled bolus (%)	14(45)	5 (26)	-		9 (45)	-	
Vallecular residuals (%)	15 (48)	6 (30)	-		9 (48)	-	
Pharyngeal-phase abnormalities (%)	7 (21)	2 (11)	-		5 (22)	-	
Aspiration (%)	3 (5)	1 (3)	-		2 (4.5)	-	

Values are expressed as the mean ± SD or number (%); Y: years; VHI: Voice Handicap Index; BECD: *Batterie Clinique d’Évaluation de la Dysarthrie* (a French adaptation of the Frenchay Dysarthria. Assessment); MPT: maximum phonation time; s: seconds; Hz: Hertz; db: decibels; F0: fundamental frequency of speech; CVF0: coefficient of variation of the fundamental frequency; DHI: Deglutition Handicap Index. Patients and controls and (within each group) men and women were compared in terms of the MPT, intensity, F0 and CVF0 (* indicates a difference between females and males,^§^ indicates a difference between PD females and control females, ^+^ indicates difference between PD males and males controls, p<0.05). The results of the deglutition assessments are presented as the percentage of individuals with a qualitative impairment, as scored in a blind manner by an experienced physician (AB or ND). The p values refer to the comparisons of men vs. women.

Seventy-four percent of the patients displayed a significant, qualitative voice alteration (UPDRS motor part item 18: score ≥1). Fifteen percent of the population presented poor speech intelligibility (UPDRS motor part item 18 ≥ 2; 80% males).

The total BECD score was also higher in men than in women, although the difference was not statistically significant (p = 0.058). Based on the subscores, hypophonia was not the main feature of dysarthria in early-stage PD; speech intonation was affected first (dysprosodia). There were no differences in qualitative speech impairments between the *de novo* patients and the treated patients.

Lastly, we compared the patients’ acoustic parameters with those of the controls. The two groups did not differ in terms of the mean maximum phonation time, voice intensity and fundamental frequency (for either gender). However, the male vs. female difference in CVF0 (a parameter reflecting dysprosodia) in the patient group (p = 0.044) differed from that found in the control group (p = 0.037). Voice tremor was present in 10 men but in only 3 women.

In order to characterize the patients’ speech further, we studied (when possible) jitter, shimmer and the harmonic-to-noise ratio (HNR). Jitter is defined as the cycle-to-cycle variation in frequency. In our PD population, the mean ± SD jitter was 0.89 ± 0.24 but did not differ significantly from the value in our control population (1.1 ± 0.21; p = 0.1). Shimmer relates to the variation in the amplitude of the sound wave. It is modified by a reduction in glottal resistance and the presence of mass lesions on the vocal cords, and is often correlated with the presence of noises and breathiness. The mean ± SD shimmer was 4.82 ±2.97. Again, this value did not differ significantly from the value found in the control group (5.94 ± 2.01; p = NS). The HNR corresponds to the ratio between periodic and non-periodic components comprising a segment of voiced speech. The periodic component arises from the vibration of the vocal cords and the non-periodic component corresponds to glottal noise (in dB). The ratio between the two components reflects the efficiency of speech, i.e. the extent to which air flow from the lungs is transformed into vibrational energy in the vocal cords. In our PD patients, the mean ± SD HNR was 18.4 ± 4.7. Although this was lower than in the control group’s value (22.4 ± 3.8), the difference was not statistically significantly (p = 0.06) and cannot be considered to be pathologic.

#### Swallowing assessments (Table 2)

Analysis of the DHI showed that the total and physical scores were low–despite the fact that few of the PD patients complained about swallowing disorders. There were no gender differences. In the glass of water test, the mean swallowing speed was significantly slower in female and male patients (p = 0.03 and 0.029; respectively) than in female and male controls.

The qualitative videofluoroscopic assessment in the “off-drug” condition revealed oral phase abnormalities in about 60% of the patients (63% of the male patients but only 30% of the female patients). Pharyngeal abnormalities were present in 21% of the patients (22% of the male patients and 11% of the female patients). Aspiration was rare (5% overall) and was observed in only 2 men and 1 woman with a PIGD phenotype.

#### Respiratory assessments (Tables 3 and 4)

On the MRC dyspnea scale, 24% of the patients reported mild dyspnea (42% of the women and 13% of the men; p<0.05) (**Table 3**). The MRC score was not significantly correlated with dysarthria (UPDRS III item 18) or speech intelligibility. In female patients, dyspnoea was significantly correlated with the HAM-A items 9 and 10 subscore (r = 0.024, p = 0.024). In male patients, dyspnoea was less frequent but more severe (when present) than in female patients, and was not related to the HAM-A subscore.

**Table 3:** The patients’ PFT results were compared with those from 36 age- and gender-matched healthy controls.

	All PD patients	Male patients	Female patients	Male controls	Female controls	p
Number of patients	66	42	24	24	12	-
Mean age	62.5 (7.95)	62.6 (7)	62.1 (8)	61 (5.2)	60.9 (4.1)	ns
BMI	26.96 (3.96)	26.76 (4.01)	26.35 (5)	-	-	ns
Self-reported dyspnea (% with MRC score >1)	24	13°	42°	-	-	°0.01
Mean HAM-A physical subscore		5.77 (5.6)°	8.69 (5.7)°	-	-	°0.001
Number with an obstructive syndrome	4	3	1	3	1	ns
Mean FEV1 (%)	105.7 (13.1)	104	105	101 (14.5)	97.6 (17)	ns
Mean FVC (%)	110.5 (14.4)	110	118	101.4 (13)	102.5(18)	ns
Mean TLC (%)	111.1 (15.7)	116.1 (16)	108 (14)	112 (12.1)	110 (14.9)	ns
Mean MIP (%)	74.8 (30.6)	76.5 (26) °	72.1 (36)*	90.61 (26) °	89 (23)*	°0.01; *0.05
Mean SNIP (%)	70.9 (28)	70.13 (67°	63 (74) *	89.7(18.6) °	88.3 (23)*	°0.0005; *0.05
Mean MEP (%)	90 (66)	105 (76)	68 (77)	102 (57)	97 (58)	ns

FEV1: forced expiratory volume in one second; FVC: forced vital capacity; TLC: total lung capacity; MIP: maximal inspiratory pressure; SNIP: sniff inspiratory pressure; MEP: maximal expiratory pressure; MRC: Medical Research Council breathlessness scale; BMI: body mass index; ns: non significant. The p values refer to comparisons of men vs. women. (°Indicates a difference between males PD and controls males, * indicates a difference between PD females and controls females, p<0.05).

**Table 4:** A comparison between patients with respiratory insufficiency (n = 22, 42%) and those with normal respiratory function (n = 38, 58%).

	Patients with inspiratory insufficiency	Patients without inspiratory insufficiency	p
N	28 (11 W/17 M)	38 (11 W/27 M)	
Age (years)	61 (8.6)	63.1 (7.4)	ns
Disease duration (years)	2 (1.4)	1.7 (1.4)	ns
MDS UPDRS part I	6.43 (2.8)	5.38 (5.0)	ns
MDS UPDRS part II	7.3 (4.4)	5.3 (4.1)	0.05
Pain subscore (out of 4)	0.9 (0.7)	0.55 (0.7)	0.005
Constipation subscore (out of 4)	0.63 (0.7)	0.28 (0.51)	0.001
UPDRS part III	23.79 (7.3)	17.8 (7.5)	0.04
Dysarthria subscore (item 18)	1.13 (0.6)	0.73 (0.6)	0.05
Axial subscore	2.9 (1.4)	2.35 (1.2)	ns
Rigidity subscore	6.27 (2.6)	4.28 (2.8)	0.03
MoCA score	27.27 (2.0)	26.50 (2.9)	ns
LARS score	-25.5 (4.7)	-27.73 (5.2)	ns
MADRS score	6.90 (5.2)	4.70 (5.1)	ns
HAM-A score	8.23 (6.1)	5.65 (5.2)	0.05
Self-reported dyspnea (%)	40	26	0.05
Swallowing disorder in the glass of water test (%)	11	5	0.05
Swallowing speed (mL/s)	14.6 (7.1)	12.71 (10)	0.05
VHI total score	30.5 (15)	22.17 (23)	0.05
DHI total score	10 (11)	11 (9)	ns
DHI physical subscore	9.5 (4.7)	5.8 (5.1)	0.05

MMSE: Mini Mental State Examination; MoCA: Montreal cognitive assessment; MADRS: Montgomery-Asberg Depression Rating Scale; LARS: Lille Apathy Rating Scale; HAM-A: Hamilton Anxiety Rating Scale; UPDRS: Unified Parkinson’s Disease Rating Scale; MDS UPDRS: Movement Disorders Society Unified Parkinson’s Disease Rating Scale; VHI: voice handicap index; DHI: deglutition handicap index; ns: non significant.

Twenty-eight patients (42%) and 9 healthy controls (25%; p = 0.008) displayed inspiratory muscle weakness. In the patient group, there were no significant correlations with age, disease duration or dopaminergic treatment, and the impairment was unresponsive to magnetic phrenic nerve stimulation in the 15 patients tested at random. Inspiratory insufficiency was significantly correlated with (i) dyspnea, (ii) severe non-motor fluctuations (especially pain and constipation), (iii) poor motor scores (especially the rigidity subscore), (iv) severe dysarthria, (v) altered swallowing speed and (vi) a higher DHI physical subscore **(Table 4).**

Lastly, there were no differences between *de novo* and treated patients in terms of the severity of respiratory, swallowing and speech disorders.

## Discussion

The present study is the first to have prospectively assessed axial symptoms in 66 consecutive early PD patients. Although the mean disease duration was only 1.22 ± 1.04 years, we observed a high frequency of axial abnormalities (namely dysarthria, qualitative swallowing impairments and inspiratory muscle weakness), and especially in men. In some patients, these impairments appeared to be interrelated. The systematic application of simple clinical tools enabled us to accurately assess these impairments. In view of the observed impact on QoL, these tools may be of value in routine clinical practice.

Our most important finding is that gender matters in PD. Women suffered more from non-motor symptoms and men presented significantly more frequent and severe axial impairments in early PD. Our results are in agreement with the recent study of 47 *de novo* drug naive PD patients by Picillo et al. [21], who found that female gender represents a major risk factor for the development of non-motor symptoms. From a practical standpoint, clinicians should take gender into account when managing PD.

Seventy-four percent of the study population displayed mild dysarthria, which affected QoL (especially in men). A recent study of 147 PD patients with a disease duration of 3.5 to 4.5 years (i.e. at a later stage in the disease than the patients in our study) also revealed a high frequency of dysphonia (in 35.4% of the patients), which was significantly associated with depression (52%) [22]. The first clinical component of dysarthria to appear in both men and women was dysprosodia, as reflected by a loss of speech intonation and a fall in CVF0. Dysphonia appeared to affect men earlier than it did women. Conversely, speech intensity and the maximum phonation time were normal; this explains the low impact of dysarthria on intelligibility in early PD. One can hypothesize that even at this stage in the disease, patients use vocal forcing to compensate for their increasing vocal problems—especially in certain bothersome situations (e.g. talking on a cellphone). Hence, dysarthria should be considered early in the course of the disease and not just as a late-stage symptom. Furthermore, clinicians definitely need new tools for the assessment of dysarthria.

The total DHI score and its subscores did not reveal significant, self-reported dysphagia. However, the glass of water test revealed marked, frequent abnormalities in swallowing in both male and female patients. In a videofluorographic assessment, 60% of the patients displayed oral-phase impairments and 21% displayed pharyngeal-phase impairments. There was no difference (relative to controls) in the total swallow duration or the frequency of aspiration (only 5% in elderly male patients with PIGD). Many studies of late-stage PD have reported significant abnormalities in deglutition (with a prevalence of between 35% and 95% [23–29]), a significant alteration in QoL, and psychosocial consequences [30,31].

Our study is the first to show that deglutition disorders are present in early-stage PD patients, regardless of the phenotype (TD or PIGD) or gender. Interestingly, the glass of water test appears to be a relevant means of detecting deglutition problems, since the results agreed with the videofluorographic assessment. Implementation of the glass of water test in routine clinical practice may be of significant value.

Respiratory problems have often been studied in heterogeneous, late-stage PD populations under a variety of experimental conditions [31]. Here, we found that 42% of the patients presented respiratory insufficiency—mainly due to a weak diaphragm. In contrast, the flow-volume curves were normal. Moreover, we detected two gender differences: women seemed more likely to suffer from dyspnea (which was linked to anxiety), whereas men were more likely to suffer from inspiratory muscle weakness. Hypokinesia and rigidity may also have contributed to these impairments.

Seccombe et al. [32] reported on abnormal respiratory control in early-stage PD; although the response to mild hypoxia was normal in most of the patients, MIP and MEP were below the normative values in 68% of cases.

Weiner et al. [33] found that in the “off-drug” condition, respiratory muscle strength and endurance were lower in PD patients than in healthy controls. The researchers also observed a non-significant trend towards higher MIP, MEP and endurance values after L-dopa intake. A central effect or the correction of uncoordinated respiratory movements by L-dopa may have contributed to this effect. In the present study, we did not find any significant differences between the *de novo* and treated subgroups–suggesting that inspiratory insufficiency (i) may be a hallmark of the pathophysiological process in PD, and (ii) is weakly influenced by dopamine agonists and levodopa (mean equivalent dose: 371 mg/day) [34,35]. However, this hypothesis will have to be confirmed.

Furthermore, the disorders caused by respiratory insufficiency may have an impact on the pathophysiology of PD. In several other neurodegenerative diseases (amyotrophic lateral sclerosis, Alzheimer’s disease and multiple system atrophy), the repeated hypoxemia caused by nocturnal breathing disorders and the desaturation caused by early-onset diaphragmatic dysfunction appear to influence the course of the disease and the cognitive outcome [36].

## Conclusion

At a time when PD is being redefined [37], we highlighted the presence of upper-body axial symptoms in prospectively included, early-stage PD patients. We notably observed that upper-body axial symptoms (i) impact QoL significantly, (ii) can be detected with a timed glass of water test, the VHI and PFT (especially dyspnea in men), and (iii) require the development of new tools for the clinician.

We suggest that upper-body axial symptoms are surrogate biomarkers for disease progression in PD (especially in men); this hypothesis must now be evaluated in large cohort studies with long-term follow-up.

## Supporting Information

S1 Table Demographical data, UPDRS scores, pulmonary function data of all the patients. (XLSX)
